# Autistic Traits and Symptoms of Social Anxiety are Differentially Related to Attention to Others’ Eyes in Social Anxiety Disorder

**DOI:** 10.1007/s10803-016-2978-z

**Published:** 2016-12-20

**Authors:** Johan Lundin Kleberg, Jens Högström, Martina Nord, Sven Bölte, Eva Serlachius, Terje Falck-Ytter

**Affiliations:** 1 Uppsala Child and Baby Lab, Department of Psychology, Uppsala University, Box 1225, Uppsala, Sweden; 2 Department of Clinical Neuroscience, Centre for Psychiatry Research, Karolinska Institutet, Stockholm, Sweden; 3 Stockholm Health Care Services, Stockholm County Council, Stockholm, Sweden; 4 Department of Women’s and Children’s Health, Center of Neurodevelopmental Disorders (KIND), Karolinska Institutet, Stockholm, Sweden; 5 Child and Adolescent Psychiatry, Center for Psychiatry Research, Stockholm County Council, Stockholm, Sweden

**Keywords:** Autism spectrum disorder (ASD), Social anxiety disorder (SAD), Attention, Eye tracking, Orienting, Avoidance, Gaze avoidance, Broader autism phenotype

## Abstract

Autism spectrum disorder (ASD) and social anxiety disorder (SAD) have partly overlapping symptoms. Gaze avoidance has been linked to both SAD and ASD, but little is known about differences in social attention between the two conditions. We studied eye movements in a group of treatment-seeking adolescents with SAD (*N* = 25), assessing SAD and ASD dimensionally. The results indicated a double dissociation between two measures of social attention and the two symptom dimensions. Controlling for social anxiety, elevated autistic traits were associated with delayed *orienting to* eyes presented among distractors. In contrast, elevated social anxiety levels were associated with faster *orienting away* from the eyes, when controlling for autistic traits. This distinction deepens our understanding of ASD and SAD.

## Introduction

Social anxiety disorder (SAD) is a psychiatric disorder characterized by intense fear of being scrutinized and negatively evaluated by others and excessive avoidance of social interaction. Autism spectrum disorder (ASD) is defined by impaired social communication and interaction alongside repetitive and restrictive behaviors causing impairment (American Psychiatric Association 2013). Previous research has demonstrated a significant overlap between symptoms of SAD and ASD. A large proportion of individuals with ASD also fulfill the criteria for SAD (Cath et al. 2007; White et al. 2009; Simonoff et al. 2008). Similarly, elevated autistic traits are prevalent in individuals diagnosed with SAD (Cath et al. 2007; Puleo and Kendall 2011). The symptomatic overlap between the conditions is mainly found in areas of social interaction and social skills, whereas restricted and repetitive behaviors and atypical social cognition may be unique to ASD (White et al. 2011). Learning disabilities and language impairments are prevalent in a large proportion of children and adolescents with ASD (Baird et al. 2006), but are typically not seen in SAD. Finally, social anxiety is more likely in older, high-functioning children and adolescents with ASD, suggesting that increased awareness of social difficulties may be a contributing factor (White et al. 2009).

It is increasingly acknowledged that both SAD and ASD can be understood as the extreme ends of continuous phenotypes, meaning that there are no clear-cut borderlines between having no impairments, elevated sub-clinical traits, and a formal diagnosis, and even among the latter group, there are substantial individual differences in symptom strength (Constantino and Todd 2005; Schneier and Socha 2010).

Individual differences in SAD symptoms in individuals with ASD may be related to atypical functioning and structure of the amygdala, a subcortical area linked to fear processing, association learning and social cognition, particularly orienting to human faces (Amaral et al. 2003; Whalen et al. 2013). In line with this, South et al. (2011) found that autonomic fear conditioning in a group with ASD was positively related to social anxiety, but negatively related to autistic traits. Taken together, this suggests that a dimensional approach is important for understanding the biological mechanisms underlying ASD.

The overlap between ASD and SAD can be attributed to a number of causes. First, it is likely that some individuals with ASD or autistic traits develop social anxiety over time, as a consequence of repeated difficulties in social interactions (Bejerot and Mörtberg 2009; White et al. 2011). Secondly, the high incidence of social anxiety in biological relatives of people with ASD suggest a degree of genetic overlap (Piven and Palmer 1999). On the other hand, there is also a concern that superficial similarities in overt behaviors result in inflated correlations between self- or parent-report targeting the two conditions (Cholemkery et al. 2014). For example, a lack of close friendship could be the result of reduced social motivation (Chevallier et al. 2012b) or a lack of social skills (Jobe and White 2007) as well as anxiety driven avoidance. An improved characterization and differentiation of the two conditions is therefore desirable.

While overlapping, there is also ample evidence showing that SAD and ASD are distinct phenomena. For example, relatives of people with a diagnosis of ASD are more likely to have signs of ASD than SAD, suggestive of a dissociable genetic background (e.g. Piven and Palmer 1999). There are also differences in phenomenology between the two conditions. Whereas repetitive behaviors and a preference for sameness are characteristic of ASD, these behaviors are rarely seen in SAD. Similarly, some individuals with ASD may prefer to be by themselves, whereas individuals with SAD typically desire social interaction (e.g. White et al. 2011; Chevallier et al. 2012a).

Both SAD and the ASD have been linked to atypicalities in social attention. In SAD, avoidance of social stimuli such as faces with direct gaze may lead to reduced opportunities to reappraise or habituate to the perceived threat. Avoidance is therefore believed to be a maintaining factor in the SAD symptomatology (Bögels and Mansell 2004). In ASD, studies have shown atypical social attention to be a precursor of clinical symptoms (Chawarska et al. 2013; Shic et al. 2014). Eye-tracking enables an analysis of attention processes that occurs at a very short timescale (within 100 ms) and have been widely used in ASD research (Falck-Ytter et al. 2013). Previous eye-tracking studies indicate that social anxiety may modulate atypical visual attention in individuals with ASD. Whereas some studies have suggested that people with ASD do not attend to others’ eyes because they don’t find them to be engaging or informative (e.g Moriuchi et al. 2016), others’ have suggested that people with ASD perceive other’s eyes as emotionally aversive, and consequently avoid looking at them (e.g Kylliäinen and Hietanen 2006). Research on social attention in ASD is complicated the fact that comorbid social anxiety is likely to be an additional influencing factor. For example, Dalton et al. (2005) found a positive relationship between amygdala reactivity and fixations on human eyes in individuals with ASD, suggesting that anxiety and aversive emotional arousal may underlie gaze avoidance in ASD. Similarly, White, Maddox and Panneton (2015) recently reported that social anxiety in a group of adolescents with ASD predicted increased attention to faces with threatening expressions. Although many questions remain, clearly studies of social attention could be important for understanding the underlying mechanisms behind ASD as well as SAD.

Eyes with direct gaze are a class of stimuli likely to be relevant for both ASD and SAD (Dalton et al. 2005; Kylliäinen and Hietanen 2006; Schulze et al. 2013). When human eyes or faces appear in the peripheral visual field, people typically orient to them with a quick gaze shift. People with ASD may prioritize facial information in the same way as typically developing populations (see Guillion et al. 2015, for a review). However, there is some evidence from eye-tracking studies suggesting that this process may be slower in people with ASD (Freeth et al. 2010; Guillon et al. 2016; Kleberg et al. 2016; Riby and Hancock 2009). Fast orienting to eyes and faces is believed to be driven by an evolutionary well preserved cortical network including the amygdala (Johnson et al. 2015).

In contrast to ASD, social anxiety and SAD have been associated with a bias to look away from potentially threatening social stimuli, including eyes with direct gaze, once they are fixated, a process likely reflecting anxiety-driven avoidance (Armstrong and Olatunji 2012; Garner et al. 2006; Schulze et al. 2013). Previous eye-tracking studies found evidence of fewer and shorter fixations to the eye region of faces in people with SAD (Horley et al. 2003, 2004; Moukheiber et al. 2010). The time course may be critical for atypical social attention in SAD and social anxiety, as avoidance has most consistently been found after the initial orienting phase (Garner et al. 2006; Wieser et al. 2009). During the earliest time period, children and adults with SAD may orient towards threat-related stimuli more rapidly than healthy controls (Armstrong and Olatunju 2012; Garner et al. 2006; Horley et al. 2004; see Waters et al. (2014) for a discussion about how this may relate to comorbid disorders). According to the vigilance-avoidance hypothesis, anxiety in social attention is best characterized by initial hyper-vigilance followed by avoidance (e.g. Garner et al. 2006).

Based on previous studies, we expected that high levels of autistic traits would be associated with slow orienting towards social stimuli, whereas high social anxiety symptoms would be related to quick orienting away from eyes once fixated (avoidance). We also examined the hypothesis that social anxiety symptoms would be associated with quicker orienting towards eyes. We tested this by studying gaze behavior during presentations of human eyes with direct gaze and nonsocial distractor images in a group of treatment seeking adolescents. All individuals in the study were diagnosed with SAD, but their symptom levels of both SAD and autistic traits varied substantially, and we could therefore assess the relation between these symptom domains and our eye-tracking measures of social attention.

## Methods

### Participants

Volunteers were recruited from an ongoing treatment study of Cognitive Behavioral Therapy (CBT) for SAD. From an initial sample of 30 adolescents, 27 participants (4 male) agreed to take part in the study. Eye tracking data was lacking from two participants due to a technical failure. Before the experiment, participants were interviewed by an experienced clinical psychologist who independently confirmed the presence of a diagnosis of SAD in all cases. Exclusion criteria were a comorbid diagnosis of ASD, psychosis, bipolar disorder, substance abuse, severe eating disorder or being a victim of domestic violence. Because of the small number of male participants, all analyses were first run in females only (*n* = 21), and then in the full sample. SPAI-C scores were lacking for one male participant. Test values are reported for the female sample. All results remained significant when male participants were included. The mean level of autistic traits as measured by raw scores in the Social Responsiveness Scale (SRS; Constantino and Gruber 2007) was 49 (*SD* = 19), which is high compared to typical populations but lower than what would be expected in children with a formal diagnosis of ASD (Bölte et al. 2008). Demographic information and clinical test scores are shown in Table 1.

**Table 1:** Participant characteristics

Measure	Female participants (*N* = 21)	Full sample (*N* = *25*)
Age	15.2 (1.2)	15.2 (1.2)
Gender proportion (female/male)	21/0	21/4
SRS total score	50 (20)	49 (19)
SRS social awareness subscale	4.8 (2.9)	4.9 (2.9)
SRS social communication subscale	15.7 (8.1)	15 (8.0)
SRS social cognition subscale	6.1 (4)	6.2 (3.9)
SRS social motivation subscale	17.4 (6.2)	17.3 (6.0)
SRS restricted interests and repetitive behaviors subscale	5.4 (3.6)	5.18 (3.7)
SPAI-C total score	35.3 (7.4)	35.0 (7.3)
Number of completed trials	8.5 (2.1)	8.1 (2.8)

Means and (standard deviations). SRS and SPAI-C values are raw scores

### Eye-Tracking Paradigm

**Fig. 1:**
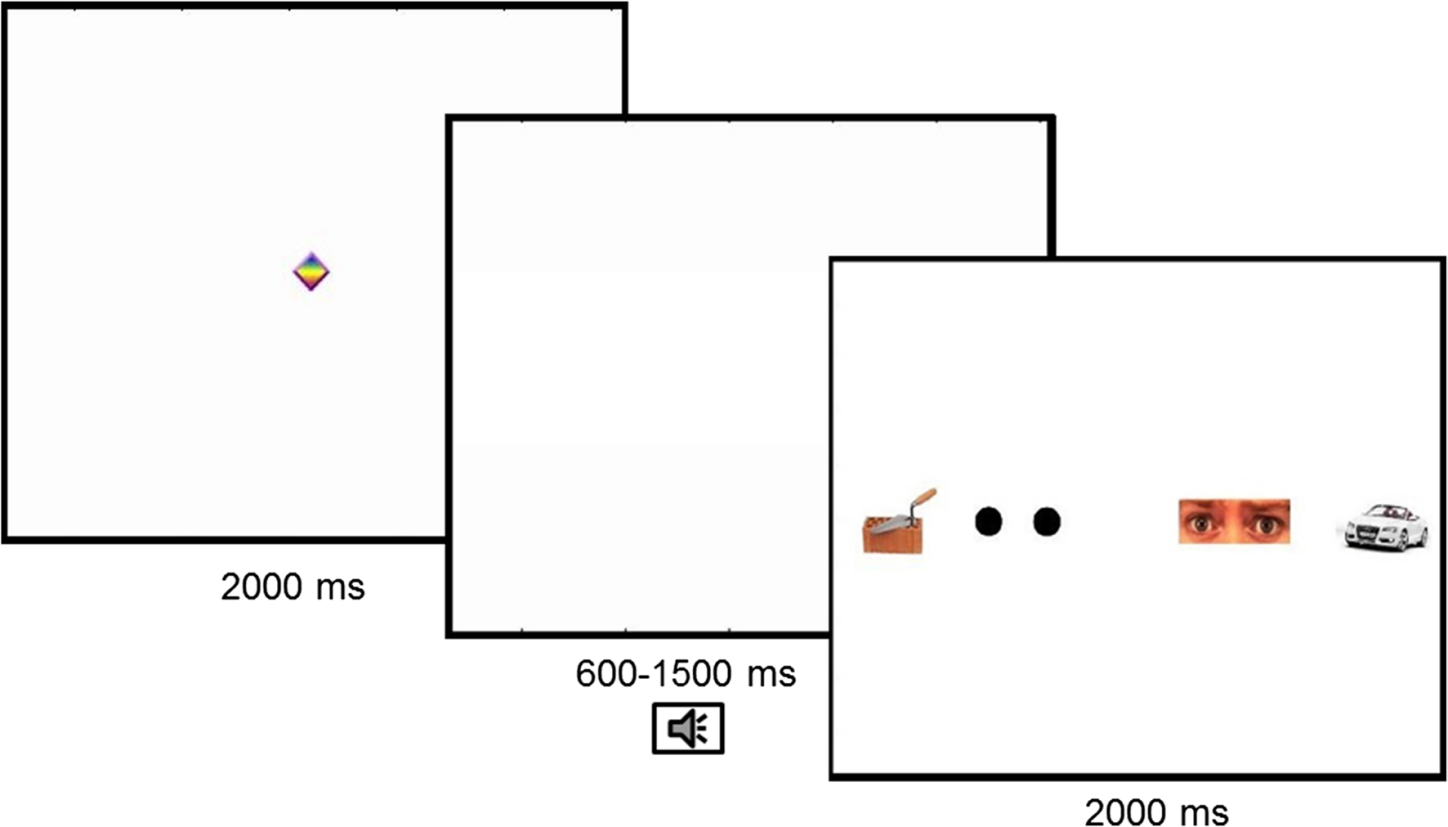
Outline of the experiment. All trials started with a brief animation at the center of the screen, followed by a blank screen during a variable time interval. A brief auditory alerting cue was presented during this interval on a subset of the trials. After this, a stimulus array with two eyes and three nonsocial objects were shown

The eye-tracking paradigm was adapted from a previous study (Kleberg et al. 2016). We presented images of human eyes with direct gaze along with three nonsocial images (Fig. 1) on a 17″ wide monitor. Stimuli consisted of 12 arrays of images (Fig. 1). In each array, one image depicted the eye region of a human face. The other three images depicted non-social objects (tools and vehicles) and a pair of black circles containing the low-level visual features of human pupils. Eye regions from the Karolinska Directed Emotional Faces (KDEF) stimulus set (Lundqvist et al. 1998) were used as stimuli. We used eyes from faces displaying fear to maximize the likelihood of orienting (Whalen et al. 2004). The stimuli were presented in randomized order. The position of stimuli within the array was counterbalanced. Eyes from 4 different faces and 12 different nonsocial objects were used as stimuli. Before each presentation, a moving animation was shown at the center of the screen to capture participants’ attention. Stimuli could be presented silently (4 trials) or preceded with by a brief auditory cue (8 trials). The auditory cues were either a single phoneme (4 trials) or a brief beep (4 trials). Auditory cues were included as an exploratory manipulation of phasic alerting. Kleberg et al. (2016) reported that phasic alerting strongly modulated social orienting in young children with ASD.

Participants were told to attend to the screen, but were given no further instructions. A nine-point calibration procedure was completed before the experiment. Data was recorded with a corneal reflection eye tracker (Tobii TX 120, Tobii Inc., Danderyd, Sweden) at a sample rate of 120 Hz.

### Clinical Measures

The Social Responsiveness Scale (SRS; Constantino and Gruber 2007) was used as a measure of autistic traits. The SRS has good psychometric properties and can be used to assess subclinical symptoms of ASD (Bölte et al. 2008). Parents completed the SRS during the visit. The Social Phobia and Anxiety Inventory—Child version (Spai-C; Beidel et al. 1998) was used as a dimensional measure of SAD symptoms. Raw scores from both measures were used.

### Data Analysis and Reduction

Fixations were identified in Tobii Studio (Tobii Inc., Danderyd, Sweden) using the Tobii Fixation Filter with distance and velocity thresholds set to 35 pixels. Further analyses were conducted in Matlab (Mathworks Inc., CA, USA) using custom scripts written by the first author. Rectangular regions of interest (ROIs) of equal size were defined around the four objects in the array. The dependent measures were (1) latency to orient to the eyes after trial onset; (2) the total fixation time inside the target ROI from first entry to first exit. The first measure indexes bottom-up driven capture of attention, whereas the latter measure is an index of extended processing.

Trials were discarded if the first fixation at target occurred quicker than 100 ms after trial onset. We also discarded trials if less than 33% of the raw samples had high quality data from both eyes (defined as Tobii studio validity codes of 0 or 1), or participants failed to look at the center of the screen for at least 50% of the baseline interval. Fixations with durations shorter than 60 ms were not analyzed.

## Results

### Preliminary Analysis

A trend towards a positive relation between the SRS and the Spai-C was found, but this was not significant [*r* (21) = .36; p = .11]. The effect of alerting cues was examined in participants with at least 50% valid trials from both the silent condition and conditions (*N* = 14). Silent trials resulted in overall longer latencies to fixate the eyes (*M* = 711; *SD* = 268) than cued trials (*M* = 611; *SD* = 173), but the difference was not significant, *t* (13) = 1278; p = .22. Trials from all conditions were therefore combined. All variables confirmed to assumptions of normality according to the Shapiro–Wilk test (all p > .35). A bivariate outlier, defined according to the Cook’s distance statistic, was removed from the analysis of the relationship between SRS and latency to first fixation at the eyes.

### Main Analysis

**Fig. 2:**
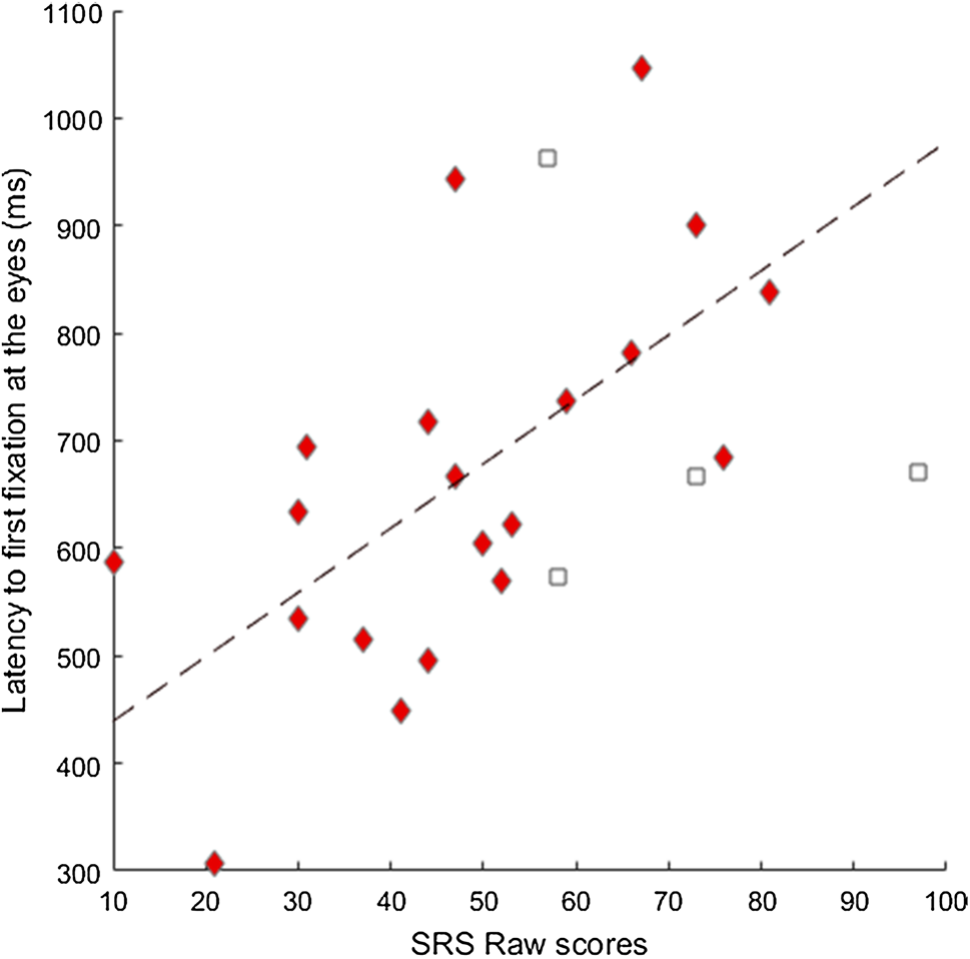
Mean latency to first fixation at the eyes plotted against SRS raw scores, with regression line superimposed. *Diamonds* represent girls. *Squares* represent boys (not included in the analysis)

A positive relation between SRS and latency to first fixation at the eyes was found, *r* = .57; p = .011, indicating that participants with higher levels of autistic traits took longer time to orient towards the eyes (Fig. 2). This relation remained when controlling for Spai-C in a partial correlation, *r* = .49; p = .04. In contrast, no significant relationship was found between Spai-C and latency to first fixation at the eyes (*r* = .35; p = .12; p = .18 after controlling for SRS).

**Fig. 3:**
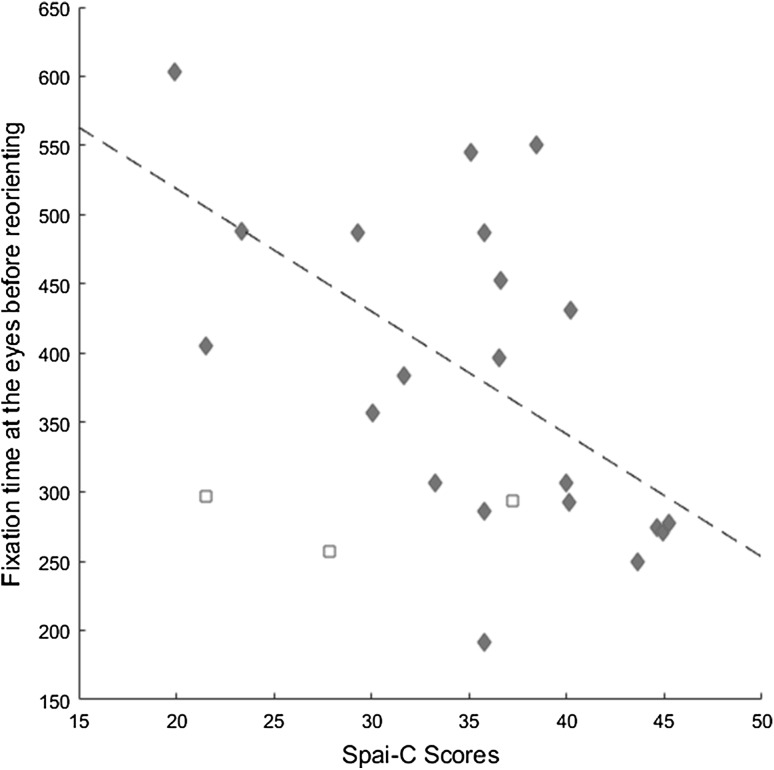
Mean fixation time at eyes before reorienting plotted against Spai-C total scores, with regression line superimposed. *Diamonds* represent girls. *Squares* represent boys (not included in the analysis)

Spai-C scores were negatively related to the time before participants first looked away from the eyes, *r* = −.58; p = .007, indicating that participants higher in social anxiety were quicker to reorient from the eyes once they were fixated (Fig. 3). This relation held when controlling for SRS, *r* = −0.56; p = .01. No relation was found between SRS and time to look away from the eyes (p = .65 after controlling for Spai-C).

The previous analyses demonstrated specific relations between autistic traits and latency to orient to the eyes on the one hand, and between social anxiety and latency to reorient from eyes on the other. However, this does not prove, for example, that the level of autistic traits is also more strongly related to the latency to orient to the eyes than social anxiety is related to the latency to orient to the eyes. In order to illuminate this issue, we compared the strength of the correlations between the symptom measures and the eye tracking variables using Steiger’s z-score comparison (Lee and Preacher 2013). The analysis showed that the association between Spai-C and the latency to look away from eyes was stronger than the association between SRS and the same eye tracking measure (*Z* = 3.097; p = .002). Using the same analysis, we did not find evidence that the SRS was more strongly related to latency to orient towards the eyes than the Spai-C (*Z* = 0.86; p = .39).

In an exploratory analysis, we calculated correlations between the eye-tracking variables and the SRS subscales (social awareness, social communication, social cognition and restricted interests and repetitive behaviors). Latency to orient to the eyes were positively related to the social communication (*r* = .47; p = .038) and social motivation (*r* = .49; p = .028) subscales, but these correlations were no longer significant when we controlled for Spai-C. In contrast, the social awareness subscale was positively related to latency to fixate the eyes only after controlling for Spai-C (*r* = .51; p = .03). Other correlations between latency to orient to the eyes and SRS subscales were not significant.

## Discussion

We found evidence of independent contributions by two aspects of social attention—orienting to and orienting away from eyes, to autistic traits and SAD symptoms, respectively. With regards to ASD, this study suggests that autistic traits are related to a reduced bottom-up driven salience of human eyes. This is generally consistent with both the impaired social orienting hypothesis (Dawson et al. 2004) and the reduced social motivation hypothesis of ASD (Chevallier et al. 2012b). In contrast, social anxiety mainly had an effect at later stages of processing, possibly reflecting anxiety-driven avoidance. As noted in the introduction, avoidance reduces the opportunity to reappraise the threat valence of social stimuli and extinguish conditioned fear responses and could therefore have a maintaining role in social anxiety (Bögels and Mansell 2004). Notably, both mechanisms operate within a very short timescale (typically within 1 s), and the distinction between them is likely to go unnoticed by the naked eye.

Social anxiety explained unique variance in terms of latency to look away from the eyes. Moreover, this relation was statistically stronger than the relation between autistic traits and the same eye tracking measure. For the latency to orient towards eyes measure the picture was less clear. Here, autistic traits did explain unique variance, but did not produce a significantly stronger correlation than anxiety. The zero order correlation between social anxiety and orienting to the eyes was not significant. Taken together, this demonstrates that autistic traits are related to delayed orienting towards eyes beyond any potential influence of social anxiety, but that questions remain regarding the relative importance of autistic traits and social anxiety for the orienting measure. While larger samples may be needed to fully understand the patterns of relations with orienting to eyes, it is important to note that our results clearly do not support the theory of initial hyper-vigilance for eyes with direct gaze in social anxiety.

Our results suggest that individual differences in the microstructure of gaze during social attention can be used to distinguish between clinically meaningful symptom dimensions in adolescents with high autistic traits and SAD (indeed, all participants had a formal diagnosis of SAD). Previous studies have demonstrated that social anxiety in populations with ASD is related to a range of clinically meaningful measures such as amygdala reactivity to faces (Dalton et al. 2005), attention to faces (Corden et al. 2008; White et al. 2015) and autonomic fear conditioning (South et al. 2011). The current study adds to this literature by demonstrating independent effects of autistic traits and social anxiety on the earliest stages of social attention—a previously unrecognized double dissociation. To our knowledge, this is the first study to examine the relationship between autistic traits and social attention in individuals with a formal diagnosis of SAD. Our results are important for understanding the differences between of ASD and SAD.

There is an ongoing debate about the causes of reduced attention to others’ eyes in people with ASD is a consequence of reduced social motivation or a form of avoidance, driven by aversive emotional reactions. Our results support the notion that autistic symptoms per se are related to reduced social motivation, whereas avoidance is best attributed to social anxiety. In line with this, a recent study of toddlers with ASD (i.e., at an age before social anxiety is likely to appear) reported evidence of reduced spontaneous attention to eyes, but no support for the avoidance account (Moriuchi et al. 2016)

Accurate identification of autistic traits in people with SAD has important clinical implications. For example, in one study, SAD patients with elevated autistic traits were found to benefit more from a form CBT with high emphasis on parent involvement than SAD patients with low-to normal levels of autistic traits (Puleo and Kendall 2011).

Using the same stimuli in a group of young children, we recently found a strong effect of auditory alerting cues in young children with ASD. Alerting cues led to faster orienting to eyes in children with ASD as compared to silent trials, whereas typically developing children showed the opposite pattern (Kleberg et al. 2016). In the present study, latency to orient to the eyes was not modulated by alerting cues I. The difference between the two studies could potentially be attributed to differences in both age and diagnosis, since the children in our previous study were both younger (with a mean age of 6.5 years) and had a formal diagnosis of ASD.

Since our sample was mainly female, further studies are needed to examine potential gender differences and generalizability to males. However, by examining a female sample, our study adds new knowledge about the broader autistic phenotype in female adolescents. Autistic symptoms may be under-recognized in girls and women, and more knowledge is needed about the symptoms and cognitive characteristics of this population (e g. Mandy et al. 2012).

The study has some limitations, including small sample size and potentially limited generalizability due to the selection criteria (treatment seeking adolescents with SAD). Nevertheless, given that the results point to a previously unknown double dissociation between two types of social attention and (dimensional) SAD and autism, the study is informative for theories of developmental psychopathology, and has potential implications for (differential) diagnosis and treatment in the future.
